# The Health Argument of Climate Action during COP26: Commentaries on Mental Health Issues

**DOI:** 10.7150/ijms.86368

**Published:** 2023-07-28

**Authors:** Fitri Fareez Ramli, Syed Alhafiz Syed Hashim

**Affiliations:** 1 Department of Pharmacology, Faculty of Medicine, Universiti Kebangsaan Malaysia, Cheras 56000, Kuala Lumpur, Malaysia.; 2 Clinical Psychopharmacology Research Group, Department of Psychiatry, University of Oxford, Oxford OX3 7JX, UK.; 3 Institute of Pharmaceutical Science, King's College London, Franklin-Wilkins Building, 150 Stamford Street, London, SE1 9NH, UK.

Climate change is a critical issue that mandates every person's participation. Various devastating impacts secondary to global warming have been reported, affecting millions of people globally. Although the physical health impact of climate change is prominent, psychological effects have also been observed. A large-scale study in the US reported an increased likelihood of negatively impacting mental health, when the maximum temperature was elevated to certain levels, and women and low-income groups had a higher probability of being affected 1. Another study in Iran had shown an increased rate of depression during summer as the minimum and maximum temperatures elevated 2. Interestingly, although the temperatures in other seasons were relatively lower than in the summer, depression prevails during autumn and winter, secondary to increased precipitation, cloudiness, and reduced sunny hours, the effects mediated by global warming 2.

Moreover, an increased prominence of psychological effects can be observed during natural disasters, with global warming causing more frequent and more climate change-related events. The psychological impact is often evident due to more severe exposure during natural disasters. One of the significant events with a substantial impact on millions of people was Hurricane Katrina which hit many parts of the US in August 2005, resulting in the increased risk of mental illness by 4% in the exposed group 1. Another recent example was the 2019/2021 Black Summer Bushfire, which affected the south-eastern regions of Australia, causing extensive exposure to smoke at hazardous levels 3. The incidence affected both physically and mentally, with reports of increased symptoms of depression or anxiety due to smoke exposure 3.

In 2015, the urgency to address the climate emergency was apparent. One-hundred-and-ninety-six countries came together in a display of solidarity for climate action, signing a legally binding international treaty known as 'The Paris Agreement' 4. The primary objective is to 'reduce global warming to well below two, preferably to 1.5 degrees Celsius than pre-industrial levels 4.' The agreement mandates that every participating Country should reduce carbon emissions with personalized plans to accomplish net-zero by 2050. Subsequently, the participating countries reconvened in Glasgow for COP26 to present the Country-specific nationally determined contributions (NDCs) and strategize the plan to reach the NDCs and protect communities and environment through collective efforts 4.

Additionally, one of the outcomes of the negotiations was the promotion of the COP26 Health Programme. The primary focus of this initiative was health professional advocacy, the establishment of a climate-resilient and sustainable health system, and promoting health adaptation research through the Adaptation Research Alliance 4. Although mental health issues were not elaborated in detail during the Health Argument of the Climate Action forum, Jagan Chapagain, the Secretary-General of the International Federal of Red Cross and Red Crescent Society, stressed the importance of mental health in relation to the climate crisis at the end of the forum. In the conclusion, he stated '… a very important aspect to look would be the impact of climate on mental health and particularly, on the young people.' Also, he expressed the approach to tackle this issue by saying, 'We have a real responsibility for today's leaders that when you are dealing with the climate crisis, while this is a big crisis, we cannot make this as the debate of hopelessness. When this becomes a debate of hopelessness, it impacts the thinking of young people massively'. He added, 'We also have to send the message of hope'. These statements could potentially enhance the awareness of mental health issues which must be translated into action in the net-zero plan.

Mental health management should not be limited to healthcare management, but must be viewed from a broader perspective. The Mayor of Utrecht, Sharon Dijksma, emphasized the importance of city planning to transform the area into a more sustainable environment, resulting in more active lifestyles and better wellbeing. According to World Bank, 56.2% of the global population reside in urban areas, indicating the importance of catering to the health of wellbeing of urban populations 5. The World Health Organization's '10 calls for Climate Action', recommends restructuring the urban environment, transportation and people mobility in efforts towards healthy, sustainable and accessible urban life 6. Greening initiative is an effort to reduce carbon footprint in various sectors, including logistics, urban planning, health care and many more. For instance, tree planting is fundamental to curbing overwhelming carbon emissions, as trees are the primary carbon sequester 7. Also, many forms of urban greening, such as parks, gardens, and forests, provide an overall cooling effect with variable magnitudes, depending on tree species and shading 8. Interestingly, the presence of green spaces is one of the determinants of mental health, as outlined by the WHO. A study in Denmark reported that exposure to green spaces during the first ten years of life has protective effects against a broad spectrum of psychiatric disorders, including depression, bipolar disorder, schizophrenia and substance abuse 9. Furthermore, a study conducted in 18 countries reported a significant positive correlation between green space exposure and mental wellbeing. Moreover, recreational visits to green and blue spaces (an accessible outdoor environment predominantly featuring water), including inland areas and coastal areas (e.g. river), were remarkably associated with higher positive wellbeing and lower mental distress 10.

In conclusion, mental health is equally important to physical health, and incorporating mental health elements is crucial in any plan related to net-zero. The governments, stakeholders, policymakers, professionals, and communities must work together to implement holistic action towards achieving net-zero goals.
